# What Is the Patient-reported Outcome and Complication Incidence After Operative Versus Nonoperative Treatment of Minimally Displaced Tibial Plateau Fractures?

**DOI:** 10.1097/CORR.0000000000003057

**Published:** 2024-05-09

**Authors:** Nick Assink, Thijs P. Vaartjes, Christiaan J. S. A. Kramer, Eelke Bosma, Robert J. Nijveldt, Joost G. ten Brinke, Reinier de Groot, Harm Hoekstra, Frank F. A. IJpma

**Affiliations:** 1 Department of Trauma Surgery, University of Groningen, University Medical Center Groningen, Groningen, the Netherlands; 2 Department of Trauma Surgery, Martini Hospital, Groningen, the Netherlands; 3 Department of Trauma Surgery, Isala Hospital, Zwolle, the Netherlands; 4 Department of Trauma Surgery, Gelre Hospital, Apeldoorn, the Netherlands; 5 Department of Trauma Surgery, Medisch Spectrum Twente, Enschede, the Netherlands; 6 Department of Traumatology, KU Leuven University Hospitals Leuven Gasthuisberg Campus, Leuven, Belgium

## Abstract

**Background:**

Much controversy remains about whether minimally displaced tibial plateau fractures should be treated operatively or nonoperatively. It is generally accepted that gaps and stepoffs up to 2 mm can be tolerated, but this assumption is based on older studies using plain radiographs instead of CT to assess the degree of initial fracture displacement. Knowledge regarding the relationship between the degree of fracture displacement and expected functional outcome is crucial for patient counseling and shared decision-making, specifically in terms of whether to perform surgery.

**Questions/purposes:**

(1) Is operative treatment associated with improved patient-reported outcomes compared with nonoperative treatment in minimally displaced tibial plateau fractures (fractures with up to 4 mm of displacement)? (2) What is the difference in the risk of complications after operative versus nonoperative treatment in minimally displaced tibial plateau fractures?

**Methods:**

A multicenter, cross-sectional study was performed in patients treated for tibial plateau fractures between 2003 and 2019 at six hospitals. Between January 2003 and December 2019, a total of 2241 patients were treated for tibial plateau fractures at six different trauma centers. During that time, the general indication for open reduction and internal fixation (ORIF) was intra-articular displacement of > 2 mm. Patients treated with ORIF and those treated nonoperatively were potentially eligible; 0.2% (4) were excluded because they were treated with amputation because of severe soft tissue damage, whereas 4% (89) were excluded because of coexisting conditions that complicated outcome measurement including Parkinson disease, cerebrovascular accident, or paralysis (conditions causing an inability to walk). A further 2.7% (60) were excluded because their address was unknown, and 1.4% (31) were excluded because they spoke a language other than Dutch. Based on that, 1328 patients were potentially eligible for analysis in the operative group and 729 were potentially eligible in the nonoperative group. At least 1 year after injury, all patients were approached and asked to complete the Knee injury and Osteoarthritis Outcome Scale (KOOS) questionnaire. A total of 813 operatively treated patients (response percentage: 61%) and 345 nonoperatively treated patients (response percentage: 47%) responded to the questionnaire. Patient characteristics including age, gender, BMI, smoking, and diabetes were retrieved from electronic patient records, and imaging data were shared with the initiating center. Displacement (gap and stepoff) was measured for all participating patients, and all patients with minimally displaced fractures (gap or stepoff ≤ 4 mm) were included, leaving 195 and 300 in the operative and nonoperative groups, respectively, for analysis here. Multivariate linear regression was performed to assess the association of treatment choice (nonoperative or operative) with patient-reported outcomes in minimally displaced fractures. In the multivariate analysis, we accounted for nine potential confounders (age, gender, BMI, smoking, diabetes, gap, stepoff, AO/OTA classification, and number of involved segments). In addition, differences in complications after operative and nonoperative treatment were assessed. The minimum clinically important differences for the five subscales of the KOOS are 11 for symptoms, 17 for pain, 18 for activities of daily living, 13 for sports, and 16 for quality of life.

**Results:**

After controlling for potentially confounding variables such as age, gender, BMI, and AO/OTA classification, we found that operative treatment was not associated with an improvement in patient-reported outcomes. Operative treatment resulted in poorer KOOS in terms of pain (-4.7 points; p = 0.03), sports (-7.6 points; p = 0.04), and quality of life (-7.8 points; p = 0.01) compared with nonoperative treatment, but those differences were small enough that they were likely not clinically important. Patients treated operatively had more complications (4% [7 of 195] versus 0% [0 of 300]; p = 0.01) and reoperations (39% [76 of 195] versus 6% [18 of 300]; p < 0.001) than patients treated nonoperatively. After operative treatment, most reoperations (36% [70 of 195]) consisted of elective removal of osteosynthesis material.

**Conclusion:**

No differences in patient-reported outcomes were observed at midterm follow-up between patients treated surgically and those treated nonsurgically for tibial plateau fractures with displacement up to 4 mm. Therefore, nonoperative treatment should be the preferred treatment option in minimally displaced fractures. Patients who opt for nonoperative treatment should be told that complications are rare, and only 6% of patients might undergo surgery by midterm follow-up. Patients who opt for surgery of a minimally displaced tibial plateau fracture should be told that complications may occur in up to 4% of patients, and 39% of patients may undergo a secondary intervention (most of which are elective implant removal).

**Level of Evidence:**

Level III, therapeutic study.

## Introduction

Tibial plateau fractures with relatively small gaps or stepoffs (up to 4 mm) might be considered minimally displaced [22]. For these injuries, operative and nonoperative management might be suitable. Gap and stepoff measurements, both representing fracture displacement, are often used clinically when deciding between operative and nonoperative treatment [12, 17, 18]. However, whether to choose operative or nonoperative treatment remains a topic of debate in patients with these minimally displaced fractures. Evidence regarding the accepted residual incongruency varies from 2 to 10 mm [6, 15, 16, 20]. For every fracture, it is important to consider whether the advantages of surgery outweigh potential complications, and this process requires shared decision-making.

However, there is very little evidence to support such a shared decision-making process in patients with minimally displaced fractures. The generally accepted indication for proceeding to surgical treatment of tibial plateau fractures includes an intra-articular fracture gap or stepoff of more than 2 mm [7, 17, 18]. This 2-mm cutoff was suggested by research that is more than 30 years old [4, 21], and to our knowledge, there is little support for the assumption that accurate articular reduction of tibial plateau fractures < 2 mm is critical to achieving a good clinical outcome [9]. In our experience, especially in minimally displaced tibial plateau fractures that slightly exceed the 2-mm threshold, there is considerable variation in treatment type across surgeons and centers. Despite the substantial impact of these injuries on patients and healthcare systems, evidence on this topic is scarce, and comparative studies between nonoperative and operative treatment of minimally displaced tibial plateau fractures are lacking. In addition, current studies are limited to residual displacement postoperatively, which does not allow for recommendations regarding how to treat fractures based on the initial displacement. A recent study demonstrated that initial fracture gaps or stepoffs up to 4 mm, as measured on CT, could result in good functional outcome after nonoperative treatment [22]; thus, it seems reasonable to revisit the old recommendation of a threshold of 2 mm based on radiographs and to see whether the threshold should be revised based on evolving insights from CT images.

We therefore asked: (1) Is operative treatment associated with improved patient-reported outcomes compared with nonoperative treatment in minimally displaced tibial plateau fractures (fractures with up to 4 mm of displacement)? (2) What is the difference in the risk of complications after operative versus nonoperative treatment in minimally displaced tibial plateau fractures?

## Patients and Methods

### Study Design and Setting

A multicenter, cross-sectional study was performed in patients treated for a tibial plateau fracture between January 2003 and December 2019 in the orthopaedic and trauma surgery department of six hospitals (four Level 1 and two Level 2 trauma centers). Hospitals consisted of two academic centers and four nonacademic centers in medium-sized cities in the Netherlands and Belgium.

### Participants

**Fig. 1:**
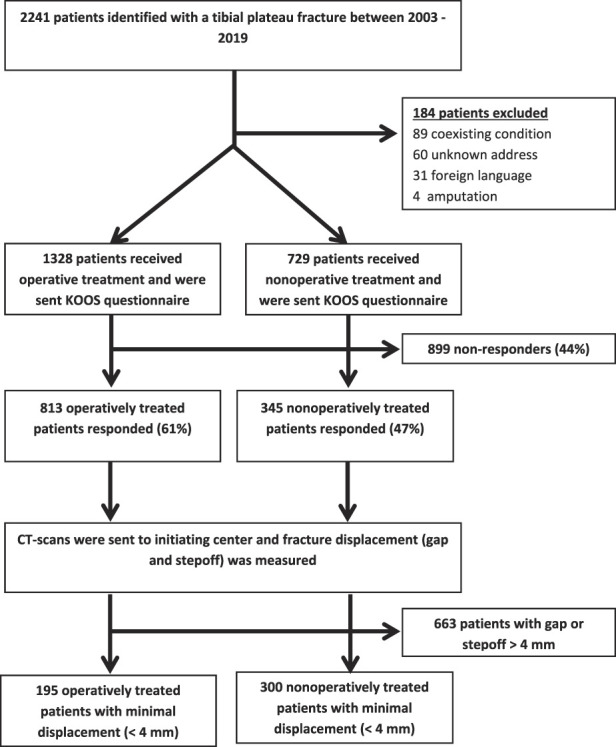
The patient inclusion process for this study is depicted in this flowchart.

Between January 2003 and December 2019, a total of 2241 patients were treated for tibial plateau fractures, had an available diagnostic CT scan, and were still alive at the time of follow-up, according to the national population registry. Patients treated with open reduction and internal fixation (ORIF) and those treated nonoperatively were potentially eligible; a total of 0.2% (4) were excluded because they underwent amputation owing to severe soft tissue damage, whereas 4% (89) were excluded because of coexisting conditions complicating outcome measurement, including Parkinson disease, cerebrovascular accident, or paralysis (that is, conditions causing an inability to walk). A further 2.7% (60) were excluded because their address was unknown, and 1.4% (31) were excluded because they spoke a language other than Dutch. Thus, 1328 patients were potentially eligible for analysis in the operative group and 729 were potentially eligible in the nonoperative group. At least 1 year after injury, all patients were approached and asked to complete the Knee injury and Osteoarthritis Outcome Scale (KOOS) questionnaire. A total of 813 operatively treated patients (response percentage: 61%) and 345 nonoperatively treated patients (response percentage: 47%) responded to the questionnaire. Patient characteristics including age, gender, BMI, smoking, and diabetes were retrieved from electronic patient records, and imaging data were shared with the initiating center. Displacement (gap and stepoff) was measured for all participating patients, and all patients with minimally displaced fractures (gap or stepoff ≤ 4 mm) were included, leaving 195 and 300 in the operative and nonoperative groups, respectively, for analysis here (Fig. 1).

### Analysis of Nonresponders

For the nonresponse analysis, we used an independent-samples t-test for continuous variables and a chi-square test for noncontinuous variables.

Overall, nonresponders did not differ very much from responders, which tends to lend credibility to the analysis. The nonresponse analysis showed that nonresponders were slightly younger than responders (51 ± 18 years versus 53 ± 16 years; p = 0.001), there was a smaller proportion of women (58% [523 of 896] versus 68% [788 of 1155]; p = 0.001), and they received nonoperative treatment more often (43% [387 of 896] versus 30% [345 of 1155]; p = 0.001). Knee radiographs and CT images of nonresponders at the initiating center and one affiliated center were the only ones available for the imaging analysis (n = 550). Analysis showed that nonresponders in these centers had smaller gaps (4.2 ± 5.2 mm versus 6.4 ± 6.3 mm; p < 0.001) and stepoff (4.5 ± 4.7 mm versus 6.3 ± 5.7 mm; p < 0.001). AO/OTA fracture classification did not differ between nonresponders and responders (B1: 11% versus 15%, B2: 16% versus 19%, B3: 45% versus 39%, C2: 6% versus 8%, C3: 22% versus 17%; p = 0.26).

### Descriptive Data

Patients who received operative treatment for minimally displaced tibial plateau fractures did not differ from nonoperatively treated patients in terms of mean ± standard deviation age (51 ± 15 years versus 54 ± 16 years; p = 0.09), gender (64% [125 of 195] versus 72% [216 of 300] women; p = 0.07), BMI (26 ± 4 kg/m^2^ versus 26 ± 5 kg/m^2^; p = 0.22), smoking (21% [40 of 195] versus 17% [50 of 300]; p = 0.38), and diabetes (7% [13 of 195] versus 8% [23 of 300]; p = 0.58) (Table 1). Operatively treated fractures consisted of split depression fractures (AO/OTA B3), whereas nonoperatively treated fractures consisted more often of pure split (AO/OTA B1) and pure depression (AO/OTA B2) fractures (p < 0.001). Additionally, operatively treated fractures had slightly more articular segments involved (4 ± 2 versus 3 ± 1; p < 0.001) (Table 1).

**Table 1.** Characteristics of patients with minimally displaced tibial plateau fractures (gap and stepoff ≤ 4 mm)

Parameter	Operative (n = 195)	Nonoperative (n = 300)	p value
Age in years	51 ± 15	54 ± 16	0.09
Women	64% (125)	72% (216)	0.07
BMI in kg/m^2^	26 ± 4	26 ± 5	0.22
Smoking	21% (40)	17% (50)	0.38
Diabetes	7% (13)	8% (23)	0.58
AO/OTA classification			< 0.001
41-B1	15% (30)	25% (75)	
41-B2	32% (62)	44% (133)	
41-B3	44% (85)	26% (77)	
41-C1	5% (9)	4% (12)	
41-C2	2% (4)	0% (1)	
41-C3	2% (5)	1% (2)	
Number of involved segments (10-segment classification)	4 ± 2	3 ± 1	< 0.001
Follow-up in years	7 ± 4	6 ± 3	0.06

Data presented as mean ± SD or % (n).

### Image Review and Gap and Stepoff Measurements

All knee radiographs and CT images that were taken at the time of injury were reassessed through consensus by an attending orthopaedic trauma surgeon (FFAIJ, > 10 years of experience) and technical physician (NA, > 5 years of experience). Fracture classification was determined according to the AO/OTA and 10-segment classification system [8, 13]. Based on fracture characteristics, the AO/OTA classification classifies intra-articular tibial plateau fractures into different types of fractures: partial articular fractures (B1: pure split, B2: pure depression, B3: split-depression) or complete articular fractures (C1: simple articular and metaphyseal, C2: articular simple and metaphyseal multifragmentary, C3: articular multifragmentary) [13]. The 10-segment classification divides the plateau into 10 segments; more involvement of segments indicate a more extensive fracture [8].

**Fig. 2:**
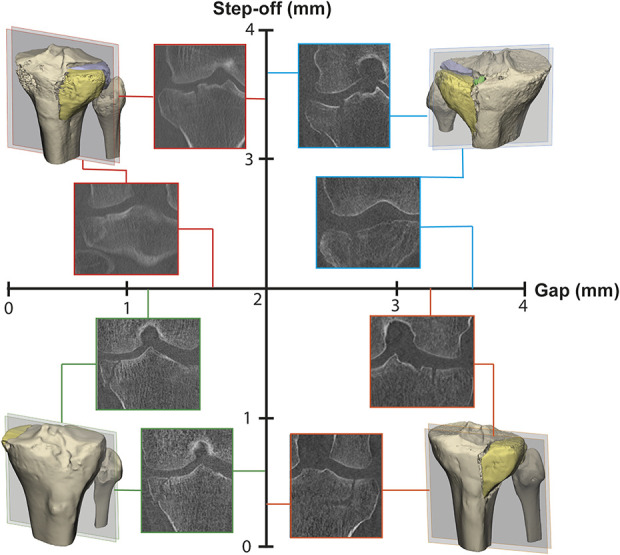
Based on their gap and stepoff, patients were divided into four groups: stepoff between 2 and 4 mm and gap less than 2 mm (upper left), gap and stepoff between 2 and 4 mm (upper right), gap and stepoff less than 2 mm (lower left), and gap between 2 and 4 mm and stepoff less than 2 mm (lower right). This illustrates the different combinations of sizes of gaps and stepoffs that are considered minimally displaced fractures.

Preoperative CT images were reassessed in the axial, sagittal, and coronal planes; in addition, gap and stepoff measurements were performed on the CT slice where displacement was found to be the highest. Gap was defined as separation of fracture fragments along the articular surface, and stepoff was characterized as separation of fracture fragments perpendicular to the articular surface [2, 22]. For each patient, the maximum value of the gap and stepoff on any of the axial, coronal, or sagittal CT slices is reported. To assess the impact of gap and stepoff separately, a subanalysis was performed in which patients were divided into four groups based on the size of their maximum gap and stepoff: patients with a gap and stepoff less than 2 mm, patients with a gap between 2 and 4 mm and stepoff less than 2 mm, patients with a stepoff between 2 and 4 mm and gap less than 2 mm, and patients with a gap and stepoff between 2 and 4 mm (Fig. 2).

### Patient-reported Outcomes

All eligible patients were approached by postal mail and asked to provide informed consent and complete the validated and standardized KOOS questionnaire in Dutch [5]. The KOOS is a questionnaire designed to assess short-term and long-term patient-relevant outcomes after knee injuries. It contains 42 items in five separately scored subscales: pain, symptoms, activities of daily living (ADL), function in sport and recreation (sports), and quality of life (QoL). We calculated subscale scores by adding the individual items (questions) and transforming scores to a range from 0 to 100, with higher scores indicating better function. The minimum clinically important differences for the five subscales of the KOOS are 11 for symptoms, 17 for pain, 18 for ADL, 13 for sports, and 16 for QOL [11].

### Data Sources

Baseline characteristics of the participants were retrieved from the patients’ electronic records. KOOS scores were calculated from the returned patient questionnaires. Gap and stepoff measurements were performed on diagnostic CT images.

### Primary and Secondary Study Goals

The primary study goal was to assess patient-reported outcomes after nonoperative and operative treatment of minimally displaced tibial plateau fractures. To assess the impact of gap and stepoff separately, a subanalysis was performed in which patients were divided into four groups with varying sizes of gap and stepoff. In addition, we performed multivariate regression to assess the association of treatment choice with patient-reported outcomes when accounting for patient characteristics and fracture characteristics (size, classification, and location). Our secondary goal was to report the incidence of complications after nonoperative or operative treatment of minimally displaced tibial plateau fractures.

### Ethical Approval

The institutional review board of all centers involved approved the study procedures, and the study was performed in accordance with relevant guidelines and regulations. All participants signed an informed consent form, and their imaging data were transferred to the initiating center.

### Statistical Analysis

The statistical analysis was performed using SPSS (version 28, IBM Corp). Differences in characteristics between operatively and nonoperatively treated patients were assessed using a t-test for continuous variables and a chi-square test for noncontinuous variables. We performed an analysis of variance test to assess the difference in patient-reported outcomes between patients in the nonoperative group and those in the operative group. We performed multivariate linear regression to assess the association of treatment choice (nonoperative or operative) with patient-reported outcomes in minimally displaced fractures. The analysis accounted for nine potential confounders (age, gender, BMI, smoking, diabetes, gap, stepoff, AO/OTA classification, and number of involved segments, according to the definition of Krause et al. [8]). Power analysis revealed that with α = 0.05%, a sample size of 126 patients (63 in the operative group and 63 in the nonoperative group) was required to provide 80% power to detect a difference of 11 points in the KOOS score (minimum clinically important difference), with a standard deviation of 20. A chi-square test was performed to assess differences in the incidence of complications and additional surgeries after operative and nonoperative treatment. A p value of less than 0.05 was considered statistically significant.

## Results

### Patient-reported Outcomes After Operative Versus Nonoperative Treatment

After controlling for potential confounding variables including age, gender, BMI, smoking, diabetes, gap, stepoff, AO/OTA classification, and number of involved segments, we found no clinically important difference between operative and nonoperative treatment in terms of all subscales of the KOOS questionnaire (Table 2, Supplemental Table 1; http://links.lww.com/CORR/B290). Operative treatment resulted in poorer KOOS score in terms of pain (-4.7 points; p = 0.03), sports (-7.6 points; p = 0.045), and QoL (-7.8 points; p = 0.01) compared with nonoperative treatment. However, this difference did not reach the point that was considered clinically important (Supplemental Table 2; http://links.lww.com/CORR/B290).

**Table 2.** Multivariate regression analysis of the association between choice of operative treatment and patient-reported outcome in minimally displaced tibial plateau fractures^a^

	β (95% CI)	p value
KOOS symptoms	-3.7 (-7.8 to 0.4)	0.08
KOOS pain	-4.7 (-9.0 to -0.3)	0.03
KOOS ADL	-3.5 (-7.7 to 0.7)	0.10
KOOS sport	-7.6 (-15.0 to -0.2)	0.045
KOOS QoL	-7.8 (-13.5 to -2.1)	0.01

The β values indicate the average increase in KOOS value associated with the choice of an operative instead of nonoperative treatment. This means that choice for operative treatment reduces the functional outcome in terms of KOOS symptoms; for example, 3.7 points compared with nonoperative treatment when accounting for confounders.

a Included confounders: age, gender, BMI, smoking, diabetes, gap, stepoff, AO/OTA classification, and involved segments.

### Complications and Additional Surgical Procedures

Patients treated operatively had more complications (4% [7 of 195] versus 0% [0 of 300]; p = 0.01) and reoperations (39% [76 of 195] versus 6% [18 of 300]; p < 0.001) than patients treated nonoperatively. After operative treatment, most reoperations (36% [70 of 195]) consisted of elective removal of osteosynthesis material (Table 3).

**Table 3.** Complications and additional surgery

	Operative (n = 195)	Nonoperative (n = 300)	p value
Complication	4% (7)	0% (0)	0.01
Fracture-related infection	2% (4)	0% (0)	
Peroneal nerve neurapraxia	2% (3)	0% (0)	
Additional surgery	39% (76)^a^	6% (18)	< 0.001
Revision for meniscal or ligamentous damage	2% (3)	3% (10)	
Corrective osteotomy after malunion	0.5% (1)	0.3% (1)	
Elective removal of osteosynthesis material	36% (70)	0% (0)	
Conversion to TKA	4% (7)	2% (7)	

Data are presented as % (n).

a Eighty-one additional surgical procedures were performed in these 76 patients.

## Discussion

Whether minimally displaced tibial plateau fractures should be treated operatively or nonoperatively remains controversial. There is no consensus regarding whether the generally used indication for operative treatment based on fracture displacement greater than 2 mm is crucial for attaining a good clinical outcome [9, 22]. We evaluated a large cohort of patients with tibial plateau fractures with minimal displacement to assess whether this recommendation based on radiographs could be justified or whether this should be revisited based on evolving insights from CT images. We found that regardless of the treatment type, no clinically important differences in patient-reported outcomes were seen at midterm follow-up. Among the patients who underwent operative treatment, 4% had a complication, and 39% of the operatively treated patients underwent one or more additional surgical procedures, often in the form of elective implant removal (36% [70 of 195]). Based on these findings, we believe that nonoperative treatment should be considered the preferred treatment option in patients with minimally displaced tibial plateau fractures with gaps and stepoffs up to 4 mm.

### Limitations

We acknowledge that this study has some degree of response bias, which is inherent to the cross-sectional study design caused by loss to follow-up and nonresponse. By approaching all patients multiple times, we reduced the risk of nonresponse bias as much as possible. In fact, our response proportion of 56% (61% for operatively treated patients and 47% for nonoperatively treated patients) is in line with what could be expected from a postal survey according to the existing evidence [10]. Nonresponse analysis showed only minor differences in terms of gender and age between responders and nonresponders, which would not affect our results. However, nonresponders were treated nonoperatively more frequently and had less displacement in terms of gap and stepoff, and it is possible that missing patients are not doing as well as those who were accounted for. Another limitation caused by the cross-sectional study design was the variation in the length of follow-up, which ranged from 1 to 17 years (mean 6 years). The consequences of gaps and stepoffs at long-term follow-up (20+ years) are still unknown. Therefore, our findings are applicable for midterm follow-up but should be interpreted with caution regarding long-term follow-up. Lastly, this study focused on fracture displacement in terms of gaps and stepoffs. However, the location and extent of the fracture might also affect the choice of operative or nonoperative treatment. By quantifying the extent of the fracture through an evaluation of the number of involved segments, as defined by Krause et al. [8], we corrected for this confounding factor in our analysis. Therefore, we do not think this limitation disqualifies our findings.

### Patient-reported Outcomes After Operative Versus Nonoperative Treatment

Regardless of the treatment type, no clinically important differences in patient-reported outcomes at midterm follow-up were found in this study. Many current guidelines on the treatment of tibial plateau fractures include an intra-articular fracture gap or stepoff of greater than 2 mm as an indication for surgical treatment [7, 17, 18]. Yet, with no expected difference in functional outcome between operative and nonoperative treatment, our results suggest that nonoperative treatment might be preferred in patients with minimally displaced fractures with initial gaps and stepoffs up to 4 mm. Much controversy remains regarding the level of articular incongruity that would be considered acceptable to justify nonoperative treatment [6]. One review of the current evidence on the correlation between residual articular fracture displacement and the risk of posttraumatic osteoarthritis concluded that the acceptable range of intra-articular stepoff should be between 2 and 10 mm [6]. By contrast, Singleton et al. [20] reported that patients with less than 2.5 mm of residual articular depression had improved outcomes. However, both studies reported on residual instead of initial fracture displacement as in our study, and therefore these results cannot automatically be translated to clinical recommendations regarding initial fracture displacement. A recent study showed that nonoperative treatment of minimally displaced fractures with initial gaps and stepoffs up to 4 mm results in good patient-reported outcomes [22]. However, this study did not directly compare nonoperatively and operatively treated patients.

One could argue that some patients with displacement exceeding the 2-mm threshold in our study might have received nonoperative treatment because of other patient-related factors such as age or comorbidities. However, this was probably not the case because patients who were treated operatively and nonoperatively in this study did not differ in terms of patient characteristics such as age, gender, BMI, smoking, and diabetes. Regarding fracture characteristics, fractures that were treated operatively seemed to involve a slightly larger area of the tibial plateau than nonoperatively treated fractures (4 ± 2 segments versus 3 ± 1 segments as defined by Krause et al. [8]). We conducted a multivariable analysis to account for these confounding factors, and it reconfirmed our findings that nonoperative treatment was noninferior to operative treatment in minimally displaced fractures with gaps and stepoffs up to 4 mm. Future studies to gain more evidence on this topic should include multicenter, prospective registry studies, preferably with preoperative and postoperative CT images and three-dimensional fracture analysis of initial and residual fracture displacement [1, 3].

### Complications and Additional Surgical Procedures

We found that operative management of minimally displaced fractures was associated with a higher chance of complications and likelihood of return to our clinic for additional surgery. Although relatively rare in minimally displaced fractures, complications such as infections and neuropraxia can severely impact patients' well-being [14]. Subjecting the patient to further surgeries introduces this risk once again. A surgeon should be thoughtful before recommending surgery, and choosing a treatment for tibial plateau fractures should involve a discussion about whether the surgical advantages outweigh the potential complications. This is especially true for minimally displaced fractures for which the advantages of surgery (such as minimal fracture reduction to reduce the risk of posttraumatic osteoarthritis) might be limited. After all, surgery is not without risks. A recent systematic review including 2214 procedures reported surgical site infections in 9.9% of patients after surgical treatment of tibial plateau fractures [19]. The minimally displaced fractures evaluated in this study had a lower infection incidence of 2%. Injury of the peroneal nerve was observed in 2% of patients. Additionally, 36% (70 patients) underwent removal of osteosynthesis material at follow-up, exposing patients to additional risks associated with surgical treatment. None of these complications were reported in the nonoperative treatment group. Although patients in the nonoperative treatment group had a number of revisions for meniscal or ligamentous damage, these numbers were lower than in the patients who had operative treatment. Reducing the risk of posttraumatic arthritis at follow-up might still be a reason to opt for operative treatment, but at midterm follow-up, we found no advantage to surgery, because there was no difference between patients treated nonoperatively and those treated operatively in terms of the proportion undergoing conversion to TKA (2% versus 4%). Future prospective studies might provide a more comprehensive overview of complications and reinterventions.

### Conclusion

Regardless of the treatment type, no differences in patient-reported outcomes were seen at midterm follow-up when patients treated surgically and those treated nonsurgically for tibial plateau fractures with displacement up to 4 mm were compared. Therefore, nonoperative treatment should be considered the preferred treatment in minimally displaced fractures. Patients who opt for nonoperative treatment should be told that complications are rare and only 6% of patients might require surgery at a later stage. Patients who choose surgery should be told that complications may occur in up to 4% of patients, and 39% of patients may undergo a secondary intervention (36% of patients an elective implant removal procedures).

## Supplementary Material

## References

[1] Assink N Bosma E Meesters AML Initial and residual 3D fracture displacement is predictive for patient-reported functional outcome at mid-term follow-up in surgically treated tibial plateau fractures. J Clin Med. 2023;12:6055. 37762994 10.3390/jcm12186055 PMC10531969

[2] Assink N El Moumni M Kraeima J Radiographic predictors of conversion to total knee arthroplasty after tibial plateau fracture surgery: results in a large multicenter cohort. J Bone Joint Surg Am. 2023;105:1237-1245. 37196070 10.2106/JBJS.22.00500

[3] Assink N Kraeima J Meesters AML 3D assessment of initial fracture displacement of tibial plateau fractures is predictive for risk on conversion to total knee arthroplasty at long-term follow-up. Eur J Trauma Emerg Surg. 2022;49:867-874. 36264307 10.1007/s00068-022-02139-y PMC10175438

[4] Brown TD Anderson DD Nepola J V Singerman RJ Pedersen DR Brand RA. Contact stress aberrations following imprecise reduction of simple tibial plateau fractures. J Orthop Res. 1988;6:851-862. 3171765 10.1002/jor.1100060609

[5] De Groot IB Favejee MM Reijman M Verhaar JAN Terwee CB. The Dutch version of the Knee Injury and Osteoarthritis Outcome Score: a validation study. Health Qual Life Outcomes. 2008;6:1-11. 18302729 10.1186/1477-7525-6-16 PMC2289810

[6] Giannoudis PV Tzioupis C Papathanassopoulos A Obakponovwe O Roberts C. Articular stepoff and risk of post-traumatic osteoarthritis. Evidence today. Injury. 2010;41:986-995. 20728882 10.1016/j.injury.2010.08.003

[7] Hall JA Beuerlein MJ McKee MD; Canadian Orthopaedic Trauma Society. Open reduction and internal fixation compared with circular fixator application for bicondylar tibial plateau fractures. Surgical technique. J Bone Joint Surg Am. 2009;91:74-88. 10.2106/JBJS.G.01165 19255201

[8] Krause M Preiss A Müller G Intra-articular tibial plateau fracture characteristics according to the “ten segment classification.” Injury. 2016;47:2551-2557. 27616003 10.1016/j.injury.2016.09.014

[9] Marsh JL Buckwalter J Gelberman R Articular fractures: does an anatomic reduction really change the result? J Bone Joint Surg Am. 2002;84:1259-1271. 12107331

[10] Meyer VM Benjamens S El Moumni M Lange JFM Pol RA. Global overview of response rates in patient and health care professional surveys in surgery: a systematic review. Ann Surg. 2022;275:e75-e81. 32649458 10.1097/SLA.0000000000004078 PMC8683255

[11] Monticone M Ferrante S Salvaderi S Motta L Cerri C. Responsiveness and minimal important changes for the Knee Injury and Osteoarthritis Outcome Score in subjects undergoing rehabilitation after total knee arthroplasty. Am J Phys Med Rehabil. 2013;92:864-870. 23900017 10.1097/PHM.0b013e31829f19d8

[12] Mthethwa J Chikate A. A review of the management of tibial plateau fractures. Musculoskelet Surg. 2018;102:119-127. 29043562 10.1007/s12306-017-0514-8

[13] Müller ME Nazarian S Koch P Schatzker J. The Comprehensive Classification of Fractures of Long Bones. Springer; 1990.

[14] Papagelopoulos PJ Partsinevelos AA Themistocleous GS Mavrogenis AF Korres DS Soucacos PN. Complications after tibia plateau fracture surgery. Injury. 2006;37:475-484. 16118010 10.1016/j.injury.2005.06.035

[15] Parkkinen M Lindahl J Mäkinen TJ Koskinen SK Mustonen A Madanat R. Predictors of osteoarthritis following operative treatment of medial tibial plateau fractures. Injury. 2018;49:370-375. 29157843 10.1016/j.injury.2017.11.014

[16] Parkkinen M Madanat R Mustonen A Koskinen SK Paavola M Lindahl J. Factors predicting the development of early osteoarthritis following lateral tibial plateau fractures: mid-term clinical and radiographic outcomes of 73 operatively treated patients. Scand J Surg. 2014;103:256-262. 24737855 10.1177/1457496914520854

[17] Pelser P. Controversies in the management of tibial plateau fractures. SA Orthopaedic Journal. 2010;9:75-82.

[18] Prat-Fabregat S Camacho-Carrasco P. Treatment strategy for tibial plateau fractures: an update. EFORT Open Rev. 2017;1:225-232. 28461952 10.1302/2058-5241.1.000031 PMC5367528

[19] Shao J Chang H Zhu Y Incidence and risk factors for surgical site infection after open reduction and internal fixation of tibial plateau fracture: a systematic review and meta-analysis. Int J Surg. 2017;41:176-182. 28385655 10.1016/j.ijsu.2017.03.085

[20] Singleton N Sahakian V Muir D. Outcome after tibial plateau fracture: how important is restoration of articular congruity? J Orthop Trauma. 2017;31:158-163. 27984441 10.1097/BOT.0000000000000762

[21] Tscherne H Lobenhoffer P. Tibial plateau fractures: management and expected results. Clin Orthop Relat Res. 1993;292:87-100. 8519141

[22] Vaartjes TP Assink N Nijveldt RJ Functional outcome after nonoperative management of tibial plateau fractures in skeletally mature patients: what sizes of gaps and stepoffs can be accepted? Clin Orthop Relat Res. 2022;480:2288-2295. 35638902 10.1097/CORR.0000000000002266 PMC9653182

