# Supplementary material for: What Is the Patient-reported Outcome and Complication Incidence After Operative Versus Nonoperative Treatment of Minimally Displaced Tibial Plateau Fractures?
Source: Clin Orthop Relat Res. 2024 May 9;482(10):1744–52. doi: 10.1097/CORR.0000000000003057 (PMC11419509; doi:10.1097/CORR.0000000000003057)
Supplement: Supplementary file 1 [file abjs-482-1744-s001.docx]

**Supplemental Table 1.** Full regression model for treatment type in relation to KOOS functional outcome

|  | KOOS-symptoms  (R2 = 0.09, adjusted R2 = 0.07) | | KOOS-pain  (R2 = 0.09, adjusted R2 = 0.07) | | KOOS-ADL  (R2 = 0.10, adjusted R2 = 0.08) | | KOOS-sport  (R2 = 0.09, adjusted R2 = 0.07) | | KOOS-QoL  (R2 = 0.10, adjusted R2 = 0.08) | |
| --- | --- | --- | --- | --- | --- | --- | --- | --- | --- | --- |
|  | β (95% CI) | p value | β (95% CI) | p value | β (95% CI) | p value | β (95% CI) | p value | β (95% CI) | p value |
| Operative rreatment | -3.7  (-7.7 to 0.4) | 0.08 | -4.7 (-9.0 to -0.4) | 0.03 | -3.5 (-7.7 to 0.7) | 0.10 | -7.6 (-15.0 to -0.2) | 0.045 | -7.8 (-13.5 to -2.1) | 0.01 |
| Age | 0.2 (0.1 to 0.4) | < 0.001 | 0.2 (0.0 to 0.3) | 0.01 | -0.1 (-0.1 to 0.1) | 0.46 | 0.1 (-0.1 to 0.3) | 0.25 | 0.3 (0.1 to 0.4) | < 0.001 |
| Male | 6.7  (2.8 to 10.5) | < 0.001 | 5.6  (1.6 to 9.7) | 0.01 | 5.1  (1.1 to 8.9) | 0.01 | 13.4  (6.5 to 20.3) | < 0.001 | 7.0  (1.7 to 12.3) | 0.01 |
| BMI | -0.8  (-1.1 to -0.4) | < 0.001 | -1.0  (-1.4 to -0.6) | < 0.001 | -1.2  (-1.6 to -0.8) | < 0.001 | -1.8  (-2.5 to -1.1) | < 0.001 | -1.4  (-1.9 to -0.9) | < 0.001 |
| Smoking | -4.8  (-9.3 to -0.2) | 0.04 | -6.4 (-11.2 to -1.6) | 0.01 | -8.0  (-12.6 to -3.2) | < 0.001 | -12.5 (-20.6 to -4.2) | 0.01 | -5.8  (-12.1 to 0.5) | 0.07 |
| Diabetes | 0.2  (-6.4 to 6.7) | 0.96 | 1.7  (-5.2 to 8.7) | 0.63 | 3.3 (-3.4 to 10.1) | 0.33 | 5.0 (-7.2 to 17.1) | 0.42 | 1.2  (-7.9 to 10.3) | 0.79 |
| Gap | 1.2  (-0.8 to 3.2) | 0.24 | 0.3  (-1.8 to 2.3) | 0.80 | 0.6  (-1.4 to 2.7) | 0.53 | 0.7  (-2.9 to 4.3) | 0.69 | -0.4  (-3.2 to 2.3) | 0.76 |
| Stepoff | 0.5  (-1.4 to 2.4) | 0.63 | 0.8  (-1.2 to 2.8) | 0.43 | 0.7  (-1.3 to 2.6) | 0.51 | 0.6  (-2.9 to 4.1) | 0.73 | 0.9  (-1.8 to 3.5) | 0.53 |
| AO/OTA | -0.03 (-2.0 to 1.9) | 0.98 | 0.8 (-1.2 to 2.9) | 0.43 | 1.3 (-0.7 to 3.3) | 0.22 | 0.4 (-3.1 to 3.9) | 0.83 | 1.7 (-1.1 to 4.4) | 0.23 |
| Involved segments | -0.5  (-1.6 to 0.6) | 0.38 | 0.5  (-1.1 to 1.2) | 0.94 | -0.5 (-1.7 to 0.6) | 0.36 | -0.3 (-2.3 to 1.8) | 0.80 | -0.4  (-1.9 to 1.2) | 0.64 |

**Supplemental Table 2.** Patient-reported outcomes after operative versus nonoperative treatment of minimally displaced tibial plateau fractures, stratified according to increasing fracture displacement

|  | Gap and stepoff < 2 mm | | Gap 2-4 mm (and stepoff < 2 mm) | | Stepoff 2-4 mm (and gap < 2 mm) | | Gap and stepoff  2-4 mm | |  |
| --- | --- | --- | --- | --- | --- | --- | --- | --- | --- |
|  | Operative (n = 28) | Nonoperative (n = 169) | Operative  (n = 36) | Nonoperative  (n = 45) | Operative  (n = 53) | Nonoperative (n = 56) | Operative  (n = 78) | Nonoperative (n = 30) | p value |
| KOOS- symptoms | 79 ± 16 | 80 ± 20 | 76 ± 21 | 86 ± 13 | 83 ± 18 | 83 ± 19 | 78 ± 23 | 85 ± 16 | 0.03^a^ |
| KOOS-pain | 78 ± 20 | 81 ± 21 | 78 ± 22 | 87 ± 17 | 85 ± 18 | 84 ± 19 | 77 ± 23 | 86 ± 18 | 0.03^a^ |
| KOOS-ADL | 82 ± 20 | 83 ± 21 | 82 ± 20 | 89 ± 17 | 87 ± 16 | 85 ± 19 | 81 ± 23 | 87 ± 18 | 0.19 |
| KOOS-sport | 54 ± 36 | 56 ± 35 | 50 ± 32 | 69 ± 32 | 56 ± 36 | 59 ± 32 | 52 ± 37 | 59 ± 35 | 0.03 |
| KOOS-QoL | 61 ± 26 | 68 ± 27 | 61 ± 27 | 72 ± 22 | 67 ± 26 | 71 ± 25 | 61 ± 32 | 74 ± 27 | 0.01^a^ |

Data presented as mean ± SD.

When stratifying groups based on the size of the gap and stepoff, operative treatment resulted in slightly lower patient-reported outcome than nonoperative treatment in terms of KOOS symptoms, pain, sports, and quality of life. A clinically important difference was only found in terms of sports in patients with a gap between 2 and 4 mm and stepoff < 2 mm.

Minimum clinically important differences: KOOS-symptoms = 11, KOOS-pain = 17, KOOS-ADL = 18, KOOS-sport = 13, and KOOS-QOL = 16

^a^  Difference unlikely to be clinically important
